# ZNF280A promotes lung adenocarcinoma development by regulating the expression of EIF3C

**DOI:** 10.1038/s41419-020-03309-9

**Published:** 2021-01-04

**Authors:** Hongsheng Liu, Yingzhi Qin, Na Zhou, Dongjie Ma, Yingyi Wang

**Affiliations:** 1 Department of Thoracic Surgery, Peking Union Medical College Hospital, Beijing, China; 2 Department of Medical Oncology, Peking Union Medical College Hospital, Beijing, China

**Keywords:** Lung cancer, Lung cancer

## Abstract

Lung adenocarcinoma (LUAD) is the most common histological subtype in non-small cell lung cancer, which is the malignant tumor with the highest mortality and morbidity in the world. Herein, ZNF280A, a member of the zinc finger protein family carrying two consecutive Cys2His2 zinc finger domains, was shown by us to act as a tumor driver in LUAD. The immunohistochemical analysis of ZNF280A in LUAD indicated its positive correlation with tumor grade, pathological stage and lymphatic metastasis, and negative relationship with patients’ survival. A loss-of-function study revealed the inhibition of LUAD development by ZNF280A in vitro and in vivo, whereas ZNF280A overexpression induced opposite effects. Statistical analysis of gene expression profiling in LUAD cells with or without ZNF280A knockdown identified EIF3C as a potential downstream of ZNF280A, which possesses similar regulatory effects on phenotypes of LUAD cells with ZNF280A. Moreover, downregulation of EIF3C in ZNF280A-overexpressed cells could attenuate neutralize the ZNF280A-induced promotion of LUAD. In summary, our study demonstrated that ZNF280A may promote the development of LUAD by regulating cell proliferation, apoptosis, cell cycle, and cell migration and probably via interacting EIF3C.

## Introduction

Lung cancer is one of the malignant tumors that seriously threaten human health and life. According to the statistics of American Cancer Society, the incidence and mortality of lung cancer rank first among all malignant tumors^1,2^. According to histopathology, lung cancer can be divided into small cell lung cancer and non-small cell lung cancer (NSCLC). Among them, the total incidence of NSCLC is ~80–85%, which can be mainly divided into adenocarcinoma, squamous cell carcinoma, and large cell carcinoma, among which lung adenocarcinoma (LUAD) is the most common type of lung cancer^3,4^. Frequently, LUAD is surrounded by many blood vessels, thus prone to metastasis and invasion^5^. The early symptoms of lung cancer are not obvious, almost no discomfort, so the vast majority of patients suffered from advanced lung cancer at the time of diagnosis, in which the lesions have occurred local invasion or distal metastasis^6^. At present, the first choice and main means of treatment for lung cancer are still surgical treatment combined with radiotherapy or chemotherapy. In recent years, although molecular targeted therapy and immunotherapy have received great attention and developed rapidly, the prognostic improvement for LUAD patients is still not ideal^7^. Therefore, exploring key regulatory molecules associated with tumor proliferation or migration is essential for understanding the molecular mechanisms of lung cancer and developing more efficient therapeutic strategies^8–10^.

Zinc finger proteins are a type of transcription factor with characteristic “finger” domains^11^. Zinc finger proteins are widely distributed in various eukaryotes, and play a function of regulating and controlling gene expression. The most significant structural feature of zinc finger protein family members is that they can generate short three-dimensional structural models of polypeptides according to their own folding patterns, and the combination with zinc ions can help them maintain the stability of this molecular structure^11^. The functional research on zinc finger proteins covers a wide range, and the existing evidence clearly points out that it has an important role in embryonic development, cell growth and differentiation, and signal transduction^12^. In recent years, there have been more and more studies on the regulatory role of zinc finger proteins in human tumors^12,13^. Multiple zinc finger proteins have been found to have a role in promoting disease development in a variety of tumors. ZNF280A was first identified in the comprehensive high-resolution genome-wide analysis of mantle cell lymphoma^14^. It carries two consecutive Cys2His2 zinc finger domains and is a member of the zinc finger protein family. However, the relationship between ZNF280A and most types of human tumors including LUAD has yet to be developed.

In this study, we explored the biological function and downstream mechanism of ZNF280A in the development and progression of LUAD. Analysis of clinical specimens revealed the potential of ZNF280A as an indicator for tumor progression and poor prognosis of LUAD patients. Loss-of-function and gain-of-function studies revealed the regulatory role of ZNF280A in the development and progression of LUAD by influencing cell proliferation, colony formation, cell apoptosis, cell cycle distribution, and cell migration. The xenografts formed by inoculation of cells with ZNF280A knockdown progress much slower relative to the control group. Furthermore, EIF3C was screened as the potential downstream of ZNF280A to mediate the regulation of LUAD development. In a word, this study identified ZNF280A as an oncogene-like factor in the development of LUAD, which may be used as an effective therapeutic target in LUAD treatment.

## Results

### ZNF280A is upregulated in LUAD tissues and expressed in LUAD cells

**Fig. 1:**
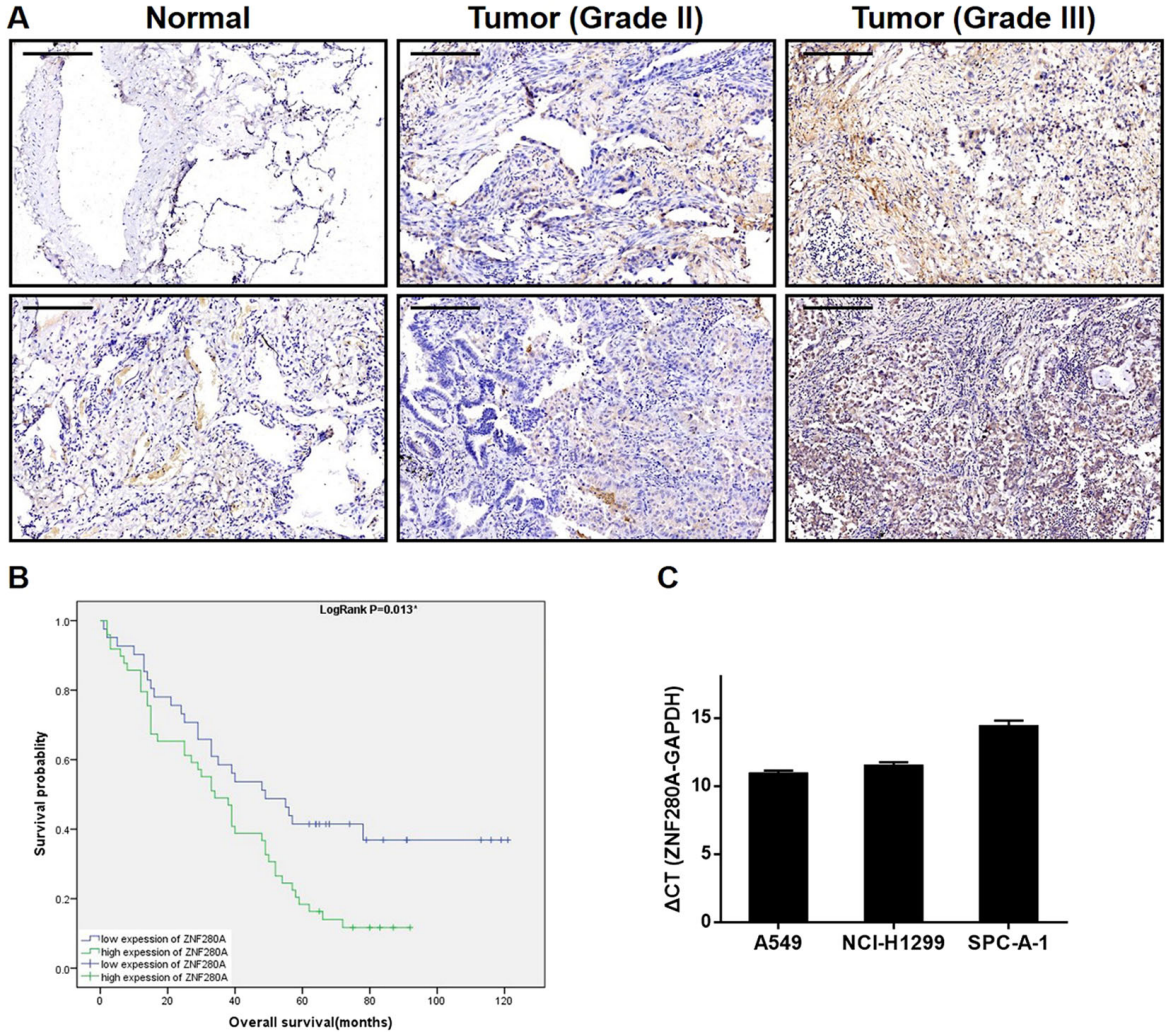
ZNF280A was upregulated in LUAD tissues and expressed in LUAD cells. **A** The expression level of ZNF280A was detected by IHC analysis in LUAD tissues and normal tissues (Scale bar = 50 μm). **B** The Kaplan–Meier survival analysis showed a significant association between ZNF280A high expression and shorter survival period of LUAD patients. **C** The mRNA expression of ZNF280A in A549, NCI-H1299, and SPC-A-1 cell lines was detected by qPCR. Data were shown as mean ± SD. *P < 0.05, **P < 0.01, ***P < 0.001.

First of all, IHC staining of 92 LUAD tissues and 70 normal tissues showed the existence of ZNF280A in LUAD tissues, and further indicated the higher level of ZNF280A in LUAD tissues relative to normal ones (Fig. 1A, Table 1). Next, we investigated the statistical correlation between ZNF280A and clinical parameters of patients with LUAD. As shown in Table 2 and Table S4, high level of ZNF280A was positively correlated with clinical characteristics including tumor grade (*P* = 0.001, Fig. 1A), pathological stage (*P* = 0.011) and lymphatic metastasis (*P* = 0.016). Moreover, Kaplan–Meier survival analysis showed the correlation between high ZNF280A expression and relatively lower survival rate (Fig. 1B).

**Table 1:** Expression patterns of ZNF280A in lung cancer tissues and normal tissues revealed in immunohistochemistry analysis.

ZNF280A expression	Tumor tissue		Normal tissue	
	Cases	Percentage	Cases	Percentage
Low	42	45.7%	70	100%
High	50	54.3%	0	/

*P* < 0.001.

**Table 2:** Relationship between ZNF280A expression and tumor characteristics in patients with lung cancer.

Features	No. of patients	ZNF280A expression		*P* value
		low	high	
All patients	92	42	50	
*Age (years)*				0.096
≤61	46	17	29	
>61	46	25	21	
*Gender*				0.169
Male	51	20	31	
Female	41	22	19	
*Tumor size*				0.077
<4 cm	39	22	17	
≥4 cm	53	20	33	
*Lymph node-positive*				0.006
<1	38	23	15	
≥1	51	16	35	
*Grade*				0.001
I	3	3	0	
II	61	33	28	
III	28	6	22	
*Stage*				0.011
1	27	18	9	
2	17	7	10	
3	42	13	29	
4	1	1	0	
*T Infiltrate*				0.038
T1	19	13	6	
T2	51	21	30	
T3	16	7	9	
T4	6	1	5	
*Lymphatic metastasis (N)*				0.016
N0	38	23	15	
N1	16	6	10	
N2	16	2	14	
N3	4	3	1	
*Expession of EGFR (FISH)*				0.944
Negative	72	34	38	
Positive	13	6	7	

*EGFR* epidermal growth factor receptor, *FISH* fluorescence in situ hybridization.

### ZNF280A knockdown inhibited LUAD development in vitro

**Fig. 2:**
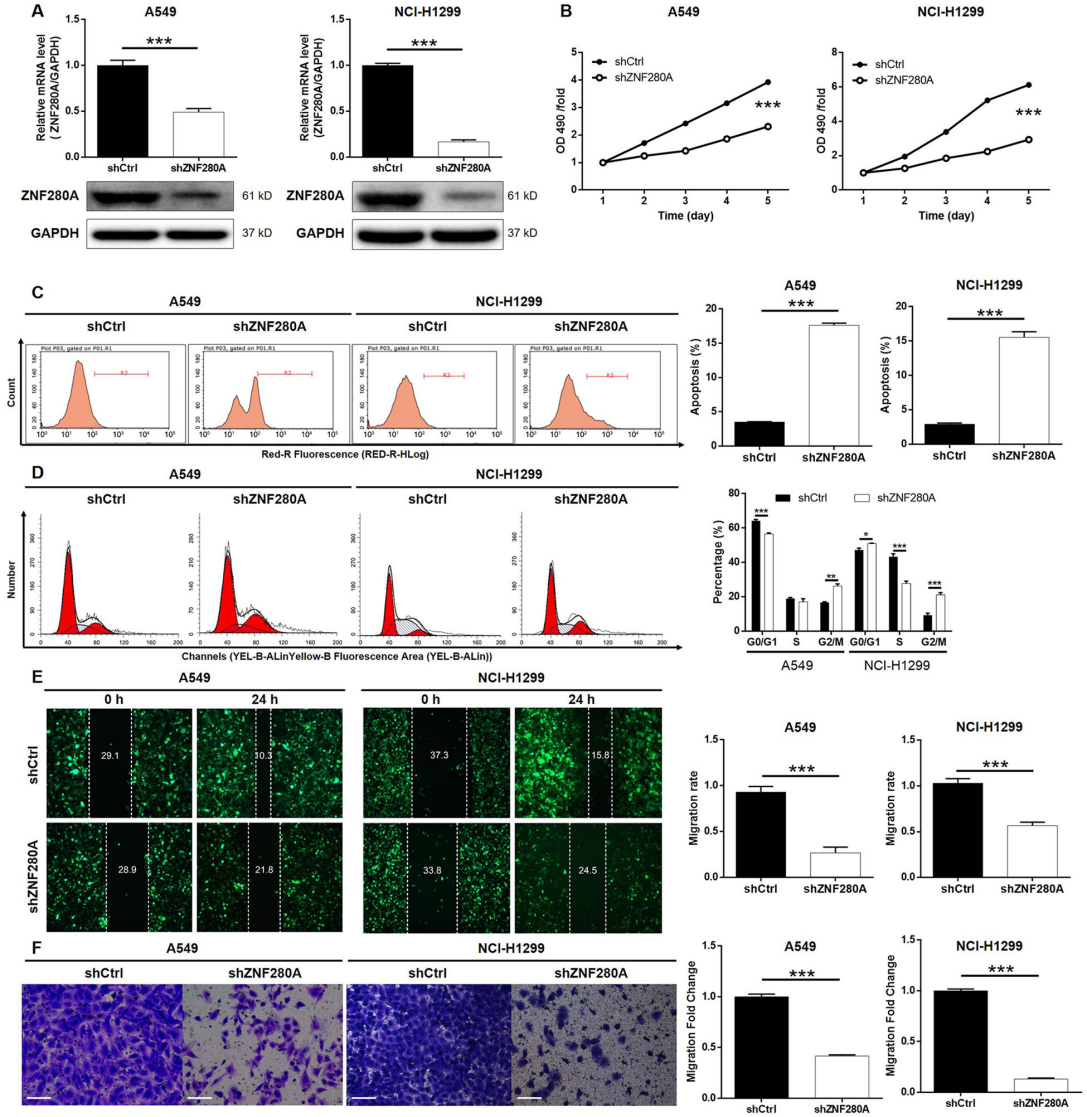
ZNF280A knockdown inhibited LUAD development in vitro. **A**, **B** Cell models with or without ZNF280A knockdown were constructed by infecting shZNF280A or shCtrl. The knockdown efficiency of ZNF280A in A549 and NCI-H1299 cells was assessed by qPCR and western blotting, respectively. **B** MTT assay was employed to show the effects of ZNF280A on cell proliferation of A549 and NCI-H1299 cells. **C** Flow cytometry was performed to detect cell apoptosis of A549 and NCI-H1299 cells with or without ZNF280A knockdown. **D** Cell cycle distribution was estimated in A549 and NCI-H1299 cells with or without ZNF280A knockdown. **E**, **F** The effects of ZNF280A on cell migration ability of A549 and NCI-H1299 cells were evaluated by wound-healing assay **E** and Transwell assay (Scale bar = 100 μm) **F**. The representative images were selected from at least three independent experiments. Data were shown as mean ± SD. *P < 0.05, **P < 0.01, ***P < 0.001.

Before carrying out in vitro studies, the expression of LUAD cell lines including A549, NCI-H1299, and SPC-A-1 was verified, among which A549 and NCI-H1299 cells with relatively higher ZNF280A expression were used as cell models. We upregulated ZNF280A expression of A549 and NCI-H1299 using shZNF280A plasmids, evaluated the transfection efficiency by fluorescence imaging (Fig. S1), and assessed the knockdown efficiency by qPCR and western blotting (Fig. 2A), respectively. 3-(4,5-dimethylthiazol-2-yl)-2,5-diphenyltetrazolium bromide (MTT) assay showed that knockdown of ZNF280A significantly inhibited LUAD cell proliferation (Fig. 2B). The results of flow cytometry indicated the enhanced cell apoptosis of shZNF280A cells, which may be resulted from the G2 phase arrest of cell cycle (Fig. 2C, D). Moreover, the expression of a series of apoptosis-related proteins in LUAD cells with or without ZNF280A knockdown was detected, showing the upregulation of Caspase3, Fas, HSP60, IGFBP-6, TNF-β, TRAILR-1, and TRAILR-2, and downregulation of Bcl-2, CD40, IGF-II, Livin, and Survivin (Fig. S2). Furthermore, the combination of wound-healing assay and Transwell assay indicated the decreased cell migration capacity in shZNF280A cells (Fig. 2E, F). All these results demonstrated the inhibition of LUAD by ZNF280A knockdown, which was consistent with the IHC results.

### ZNF280A knockdown inhibited tumor growth of LUAD in vivo

**Fig. 3:**
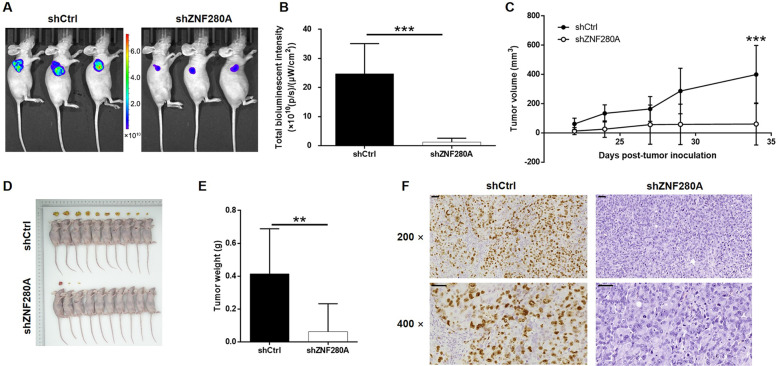
ZNF280A knockdown inhibited LUAD development in vivo. **A** 22 days post injection of NCI-H1299 cells with or without ZNF280A knockdown, the volume of tumors formed in mice was measured and calculated at indicated time intervals. **B** In vivo imaging was performed to evaluate the tumor burden in mice of shZNF280A and shCtrl groups at day 34 post tumor inoculation. **C** The bioluminescence intensity was scanned and used as a representation of tumor burden in mice of shZNF280A and shCtrl groups. **D**, **E** Mice were killed at day 34 post injection, and the tumors were removed for collecting photos **D** and weighing E. **F** The expression of Ki67 in sections of xenografts was detected by IHC analysis (Scale bar = 50 μm). Data were shown as mean ± SD. *P < 0.05, **P < 0.01, ***P < 0.001.

For in vivo study, subcutaneous xenografts were established through injection of NCI-H1299 cells with or without ZNF280A knockdown. The observations of in vivo fluorescent imaging and tumor growth curve showed that shCtrl tumors grew more rapidly than the shZNF280A tumors (Fig. 3A, B). The larger size and weight of shCtrl tumors were also verified after collecting xenografts after sacrificing mice (Fig. 3C, D). Consistently, ZNF280A insufficiency induced the lower expression of Ki67, as well as lower proliferative activity, in shZNF280A tumors (Fig. 3E). The outcomes of in vivo experiments were also in agreement with the previously mentioned results, indicating the inhibition of LUAD by ZNF280A knockdown.

### The potential of EIF3C as the downstream of ZNF280A in the regulation of LUAD

**Fig. 4:**
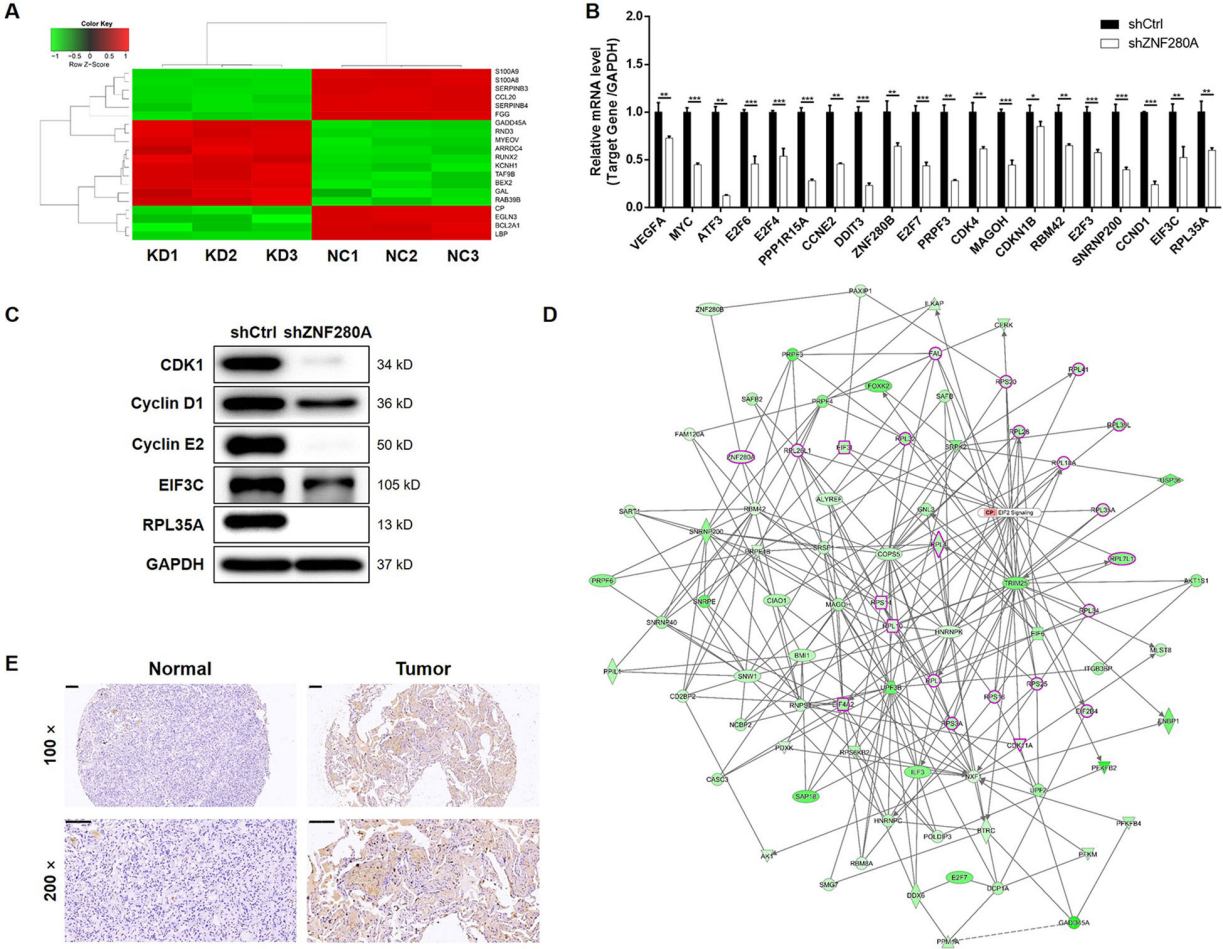
The exploration and verification of downstream underlying ZNF280A-induced regulation of LUAD. **A** A PrimeView Human Gene Expression Array was performed to identify the differentially expressed genes (DEGs) between shZNF280A and shCtrl groups of NCI-H1299 cells. **B**, **C** qPCR **B** and western blotting **C** were used to detect the expression of several selected DEGs in NCI-H1299 cells with or without ZNF280A. **D** A ZNF280A associated interaction network constructed by IPA analysis revealed the potential linkage between ZNF280A and EIF3C. **E** The expression of EIF3C in LUAD tissues and normal tissues was evaluated by IHC analysis (Scale bar = 100 μm). Data were shown as mean ± SD. *P < 0.05, **P < 0.01, ***P < 0.001.

For further insight into the mechanism by which ZNF280A regulates LUAD, microarray analysis of NCI-H1299 cells with or without ZNF280A identified 6250 differentially expressed genes (DEGs), including 2735 upregulated ones and 3515 downregulated ones (Fig. 4A, S3A, B). Accordingly, the enrichment of all DEGs in canonical signaling pathways and disease/function was interpreted by IPA, showing EIF2 signaling as one of the most enriched pathways and cancer as the most enriched disease (Fig. S3C, D). Subsequently, a variety of top-ranked DEGs was subjected to qPCR and western blotting for assessing the difference of expression induced by ZNF280A (Fig. 4B, C). Further combining the molecular interaction network constructed centered by ZNF280A and based on all above results, EIF3C was regarded as the most promising downstream of ZNF280A (Fig. 4D). As expected, IHC analysis revealed the upregulation of EIF3C in LUAD tissues (Fig. 4E).

### Knockdown of EIF3C blocked development of LUAD in vitro

**Fig. 5:**
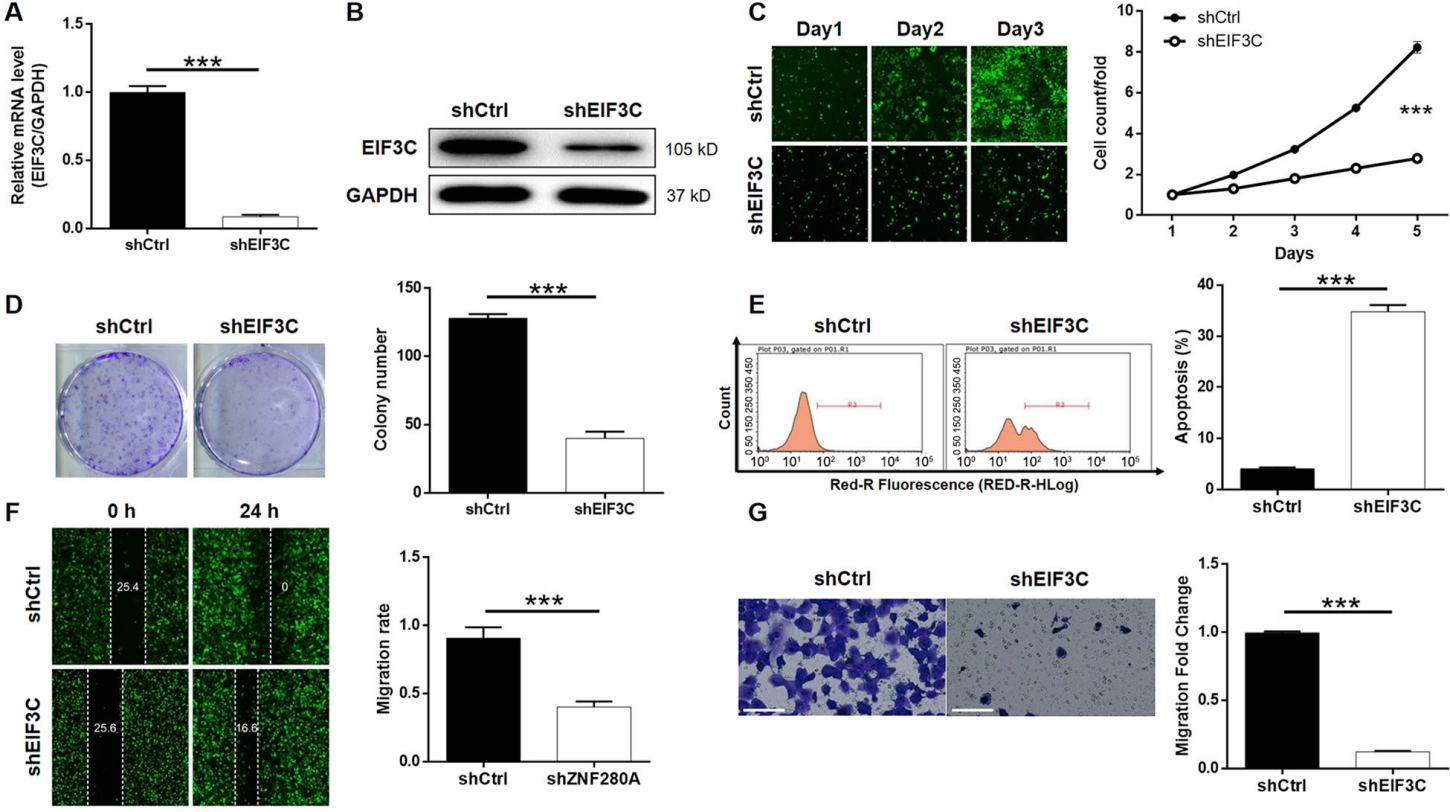
EIF3C knockdown inhibited LUAD development in vitro. **A**, **B** Cell models with or without EIF3C knockdown were constructed. The knockdown efficiency of EIF3C in NCI-H1299 cells was assessed by qPCR **A** and western blotting **B**, respectively. **C** Celigo cell counting assay was employed to show the effects of EIF3C on cell proliferation of NCI-H1299 cells. **D** Colony formation assay was used to evaluate the ability of NCI-H1299 cells with or without EIF3C knockdown to form colonies. **E** Flow cytometry was performed to detect cell apoptosis of NCI-H1299 cells with or without EIF3C knockdown. **F**, **G** The effects of EIF3C on cell migration ability of NCI-H1299 cells were evaluated by wound-healing assay **F** and Transwell assay (Scale bar = 100 μm) **G**. The representative images were selected from at least three independent experiments. Data were shown as mean ± SD. *P < 0.05, **P < 0.01, ***P < 0.001.

Using similar strategy with the above-mentioned in vitro study, lentivirus expressing the most efficient EIF3C-targeting shRNA was successfully transfected into NCI-H1299 cells to conduct loss-of-function study of EIF3C (Fig. S4). As anticipated, upon the downregulation of endogenous EIF3C expression in NCI-H1299 cells (Fig. 5A, B), Celigo cell counting assay showed the inhibited cell proliferation (Fig. 5C); colony formation assay indicated the less-formed colonies (Fig. 5D); flow cytometry demonstrated an 8.5-fold elevation of cell apoptosis rate (Fig. 5E); wound-healing and Transwell assays suggested the suppressed migration capability (Fig. 5F, G). The similar effects of ZNF280A knockdown and EIF3C knockdown implied that ZNF280A could be a positive regulator of EIF3C, thus promoting LUAD.

### EIF3C knockdown alleviated ZNF280A overexpression induced promotion of LUAD

**Fig. 6:**
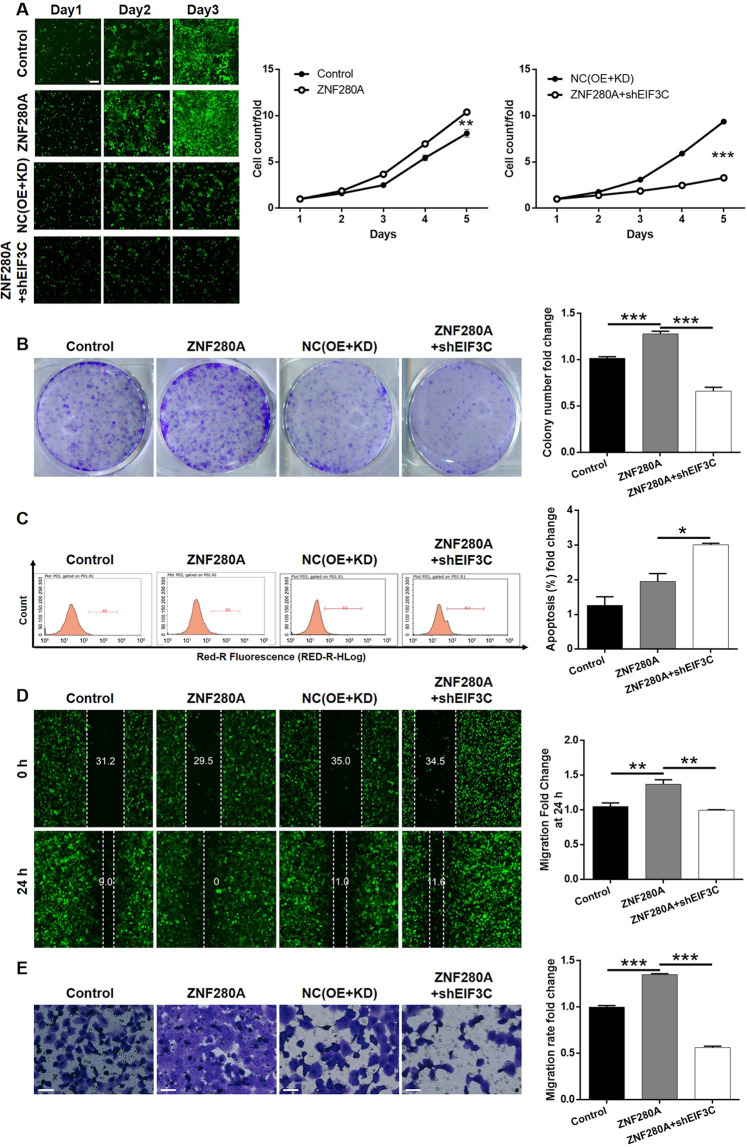
Knockdown of EIF3C attenuated the effects of LUAD cells by ZNF280A overexpression. NCI-H1299 cells transfected with Control plasmids, ZNF280A overexpression plasmids, NC(OE + KD), and simultaneous ZNF280A overexpression plasmids and shEIF3C were subjected to the detection of cell proliferation by Celigo cell counting assay (Scale bar = 400 μm) **A**, colony formation **B**, cell apoptosis by flow cytometry **C**, cell migration by wound-healing assay D and cell migration by Transwell assay (Scale bar = 50 μm) **E**. The representative images were selected from at least three independent experiments. Data were shown as mean ± SD. *P < 0.05, **P < 0.01, ***P < 0.001.

In order to further clarify the effects of ZNF280A/EIF3C axis in the regulation of LUAD, ZNF280A overexpression plasmids were used alone for constructing ZNF280A-overexpressed cell group (ZNF280A group, Fig. S5A for transfection efficiency, Fig. S5B, C for overexpression efficiency), or together with shEIF3C for constructing cell group with simultaneous ZNF280A overexpression and EIF3C knockdown (ZNF280A + shEIF3C group, Fig. S6A for transfection efficiency, Fig. S6B, C for expression detection). As shown in Fig. 6, except for the unexpected regulation of cell apoptosis, ZNF280A overexpression promoted the development of LUAD through accelerating cell proliferation (Fig. 6A), enhancing colony formation (Fig. 6B) and restraining cell migration (Fig. 6D, E). On the other hand, the overall inhibitory effects of cell growth and colony formation (Fig. 6A, B), increase of cell apoptosis (Fig. 6C) and suppression of cell migration (Fig. 6D, E) declared that the ZNF280A expression induced influence on LUAD development could be attenuated or reversed by EIF3C, highlighting the role of ZNF280A/EIF3C axis in LUAD.

## Discussion

The occurrence of LUAD is a slow and long-term complex process involving multi-factors, multi-gene regulation, and network imbalance, which ultimately leads to cell canceration. Although people’s understanding of the pathogenesis of lung cancer is not complete, it is not completely unrecognizable. The occurrence and development of tumors mostly result from a series of abnormalities at the molecular level, including mutations or amplification of some genes, abnormal activation or inactivation of signaling pathways, and imbalances in the homeostasis of the cellular environment^15^. A large number of molecules and related signaling pathways in cancer cells have crucial roles in the biological phenotype of LUAD^4^. Therefore, in recent years, more and more studies have focused on exploring the genes related to the proliferation and migration of LUAD and elucidating their regulatory mechanisms, so as to promote the understanding of the molecular mechanism of the occurrence and development of LUAD^16,17^.

Herein, we recognize ZNF280A, a zinc finger protein with C2H2 domain, as a potential tumor promotor and prognostic indicator in LUAD. High expression of ZNF280A in LUAD tumor tissues was significantly correlated with the advanced grade of tumors and could forecast relatively shorter survival period. The interference of endogenous expression of ZNF280A in LUAD cells suppresses cell growth and induces cell apoptosis through arresting cell cycle in G2 phase. LUAD cells with lower expression of ZNF280A implanted in nude mice also showed significantly decreased in vivo growth. Moreover, ZNF280A knockdown was also expounded to influence migratory capacity of LUAD cells, highlighting its potential role in tumor metastasis. On the other hand, ectopic expression induces converse effects on malignant phenotype of LUAD cells.

Zinc finger protein refers to a class of protein that contains short, stable, self-folding “finger” structures by binding zinc ions. Owing to its own structural characteristics, it can selectively combine with specific target structures, making zinc finger proteins a critical participant in gene expression regulation, cell differentiation, embryonic development and other life processes^18^. According to the difference of conserved domains, zinc finger proteins can be divided into Cys2/His2-type (C2H2), C3H, C3HC4, C2HC5, C4HC3, C2HC, C4, C6, and C8 subfamilies^19^. Among them, C2H2-type zinc finger proteins have been identified as critical regulators in the development and progression of various human cancers. For example, Jen et al.^20^ identified C2H2-type zinc finger protein ZNF322A as a transcriptional inhibitor of c-Myc, thus maintaining lung cancer stem cell-like properties by altering metabolism towards oxidative phosphorylation. Zhang et al.^21^ found that ZNF687 with C2H2 domain could enhance tumor development and promotes recurrence of hepatocellular carcinoma through transcriptional regulating BMI1, OCT4, and NANOG. On the other hand, although ZNF280A contains two consecutive C2H2-type zinc finger domain transcription factors, there are few studies on its tumor biology. The comprehensive high-resolution whole-genome analysis revealed that ZNF280A is homozygous for deletion in mantle cell lymphoma^14^. Wang et al.^22^ reported the tumor promotion effects of ZNF280A in the development of colorectal cancer, which is resulted from the activation of Hippo signaling.

In order to explore the downstream of ZNF280A in the regulation of LUAD development, gene expression profiling of LUAD cells with or without ZNF280A was obtained, based on which bioinformatics analysis was performed. It was thus deduced that ZNF280A may act as a tumor driver in LUAD by influencing EIF3C expression. A “rescue” test on phenotype regulation of ZNF280A together with EIF3C knockdown revealed EIF3C as a essential mediator in the ZNF280A-induced promotion of LUAD.

The process of eukaryotic translation can be divided into four steps: initiation, extension, termination, and recycling. Translation regulation is mainly realized in the initial stage, which is regulated by 12 known eukaryotic initiation factors (eIFs), which is the speed limiting step in the whole process. In the past decades, the functions of eIFs in the development and progression of malignant tumors have attracted more and more attention^23^. For example, a recent report demonstrated that treatment of ER^+^ breast cancer and *KRAS*-mutant NSCLC cells with eIF4A inhibitor could inhibit cell cycle feedback response and drug-resistance to CDK4/6 inhibitor treatment^24^. Moreover, the role of eIFs such as eIF6 in lung cancer was also revealed to some extent^25^. eIF3 is the largest and most complex eukaryotic translation initiation factor. It is one of the central factors in the protein translation initiation complex that interact with other translation initiation factors. At present, it is well-known that eIF3 consists of 13 subunits, which is named eIF3a to eIF3m. Research on eIF3 is mostly concentrated in malignant tumors. In recent years, studies have found that some eIFs subunits are abnormally expressed in tumor cells, and the abnormal expression of these subunits is related to the malignant behavior of tumors^26^. It has been illustrated that some subtypes of eIF3 can affect the prognosis of tumor patients by affecting the proliferation, activation, and apoptosis of tumor cells^27^. For example, EIF3A, the largest subunit of EIF3 family, has been found to be abnormally overexpressed in a variety of malignant tumors, which may be a promising therapeutic target for the design of anti-cancer drugs^28^. EIF3C was also demonstrated to participate in the regulation of human cancers such as ovarian cancer^29,30^, renal cell carcinoma^31^, osteosarcoma^32^, and cervical cancer^33^.

In conclusion, a novel LUAD tumor promotor, ZNF280A, was identified. Upregulation of ZNF280A in LUAD was associated with higher level of malignancy and poorer prognosis. Knockdown of ZNF280A inhibits LUAD development in vitro and in vivo. The mechanistic study proposed that ZNF280A may promote LUAD progression through EIF3C. Generally speaking, ZNF280A/EIF3C axis has a crucial role in LUAD, which may be promising targets for LUAD treatment.

## Materials and methods

### Cell lines and cell transfection

A549 and NCI-H1299 cells were purchased from the Cell Bank of Type Culture Collection of Chinese Academy of Sciences and NCI-H1299 were cultured in RPMI-1640 medium (Gibco) with 10% fetal bovine serum (FBS) and A549 was maintained in McCoy’s 5A Medium with 10% FBS. All cells were cultured in a humidified cell culture incubator at 37 °C under 5% CO_2_ with culture medium changed every 72 h.

For stable gene expressing, lipofectamine RNAimax (Cat. #13778075, Thermo fish) were used for cell A549 and NCI-H1299 transfection with lentiviral plasmids collected. Cells were harvested after 72 h culturing, and cell infection efficiency was valued with LV-shCtrl cells as control.

### IHC staining

Human lung cancer and para-normal tissue chip (Cat. #HLugA180Su05, Shanghai Outdo Biotech Company) was used and patients’ information was collected. For IHC staining, deparaffinized and rehydrated tissue sections were blocked and incubated with primary antibody ZNF280A (Cat. #bs-12839R, BIOSS) and followed incubated by secondary antibody. DAB color was developed with diaminobenzene and hematoxylin. Slides were pictured with microscopic and viewed with ImageScope and CaseViewer. All slides were examined randomly by two independent pathologists and IHC outcomes were determined by staining percentage and intensity scores. Staining percentage scores were classified as: 1 (1–24%), 2 (25–49%), 3 (50–74%), and 4 (75%-100%). Staining intensity were scored 0 (Signalless color) to 3 (light yellow, brown and dark brown). Antibodies used in IHC were listed in Table S1.

### RNA interference and plasmids packaging

shRNA sequences targeting human ZNF280A and EIF3C gene were designed and cDNAs were synthesized by Shanghai Yibeirui Bioscienceres, Co., Ltd. and subsequently cloned into luciferase-labeled BR-V-108 vector. In addition, ZNF280A was amplified and cloned into the BR-V112 vector after double digestion by BamHI and AgeI, and sequenced. Lentiviral particles were collected, following co-transfection using pHelper 1.0 and pHelper 2.0 vector for plasmids packaging. The sequences used were listed in Table S2.

### RNA extraction and RT-qPCR

After 72 h for ZNF280A and/or EIF3C RNA expressing, A549 and NCI-H1299 cells in triplicate were fully lysed and total RNA was extracted using TRIzol reagent (Sigma). The RNA quality was evaluated by Nanodrop 2000/2000C spectrophotometer (Thermo Fisher Scientific). cDNA was reversely transcribed from RNA using Promega M-MLV Kit (Promega) and qPCR was performed with SYBR Green mastermixs Kit (Vazyme) by applying Biosystems 7500 Sequence Detection system. Glyceraldehyde 3-phosphate dehydrogenase (GAPDH) was acted as inner control, and the primers used for the PCR reaction were showed in Table S3. The relative quantitative analysis in gene expression data were analyzed by the 2^−ΔΔCt^ method.

### Western blotting, co-immunoprecipitation, and human apoptosis antibody array

Cells were lysed in ice-cold radioimmunoprecipitation assay buffer (Millipore), and the protein were collected and the concentration was detected by a BCA Protein Assay Kit (HyClone-Pierce). Protein samples (20 μg per lane) were separated by 10% sodium dodecyl sulfate polyacrylamide gel electrophoresis (Invitrogen), and transferred onto polyvinylidene difluoride membranes at 4 °C. The membranes were blocked with tris-buffered saline tween-20 solution of 5% degreased milk at room temperature for 1 h and incubated with primary antibodies and GAPDH antibodies at 4 °C overnight. Then the membranes were incubated with secondary antibody HRP goat anti-rabbit IgG for 2 h at room temperature. The blots were visualized by enhanced chemiluminescence (Amersham).

For Human Apoptosis Antibody Array, briefly, 20 µg total proteins were cultured with the antibody-coated array membranes and then continuing incubated with HRP linked Streptavidin conjugate.

All the antibodies used in western blotting were listed in Table S1.

### Cell proliferation analysis

The cell viability was determined by MTT assay, briefly, transfected A549 and NCI-H1299 cells were stained with MTT reagent (5 mg/mL, GenView) and Formazan was dissolved by DMSO solution. The absorbance values at 490 nm were measured by microplate reader (Tecan) and the reference wavelength was 570 nm.

Cell proliferation rate was analyzed by Celigo cell counting assay. In brief, targeting cells were seeded at a 96-well plate with 2000 cells per well. The plate was continuously detected by Celigo (Nexcelom) for 5 days at the same time.

For colony formation assay, cells in the logarithmic growth phase were seeded into 6-well plates in triplicate and further cultured for 8 days. Cell clones were fixed with 4% paraformaldehyde and stained with Giemsa. Then clones were photographed under a fluorescence microscope (Olympus) and colony number (clone contains >50 cells) was counted.

### Cell apoptosis and cells cycle assay

The flow cytometric methods of identifying apoptotic cells was applied using Annexin V-APC Apoptosis kit (Cat. #88–8007, eBioscience). For cells cycle assay, cells were stained with 1 mL propidium iodide (PI) staining solution (40× PI, 2 mg/mL: 100 × RNase, 10 mg/mL: 1× PBS = 25:10:1000). FACScan and FlowJo 7.6.1 (Ashland) was used for analyze. Cell apoptosis was measured and the percentage of the cells in G0-G1, S, and G2-M phase were counted and compared.

### Cell migration assays

In order to analyze the migration ability of transfected cells in our research, wound-healing assay and transwell assay were performed. For wound-healing assay, lentivirus transfected A549 and NCI-H1299 cells (5 × 10^4^ cells/well) were plated into 96-well plates for culturing. Scratches were made by a 96 wounding replicator (VP scientific). Photographs were taken by a fluorescence microscope at 0 h, 8 h, and 24 h and cell locations were recorded, respectively. Cell migration rates of each cell group were calculated. In transwell assay, cells were seeded into a 24-well plate in the upper chambers, and medium supplemented with 30% FBS was added into the lower chambers. Cells were fixed with 4% formaldehyde and stained by Giemsa and the migration ability of cells was analyzed.

### Microarray analysis

Total RNA from NCI-H1299-shCtrl and NCI-H1299-shZNF280A cells was extracted using TRIzol. RNA quantity and quality were assessed with a Thermo Nanodrop 2000 (1.7 < A260/A280 < 2.2, Thermo Fisher Scientific). Affymetrix PrimeView Human Gene Expression Arrays (Thermo Fisher Scientific) were used for microarray analysis to obtain gene expression profiles according to the manufacturer’s instructions. Significantly DEGs were selected based on *P* < 0.05 and |Fold Change | > 1.3. KEGG pathway enrichment analysis was performed for all significant DEGs.

### Animal experiments

All animal studies were approved by Ethics committee of Peking Union Medical College Hospital. Female BALB/c nude mice were purchased from Shanghai Lingchang Experimental Animals Co., Ltd. For tumorigenicity, 5 × 10^6^ lentivirus (shCtrl or shZNF280A) transfected NCI-H1299 cells were subcutaneously injected into each mouse (4-week-old, *n* = 10 per group). Mice’s weight and tumor sizes were recorded two times per week and the volume of tumor was calculated as *π*/6 × *L* × *W*^2^ (*W*, width at the widest point; *L*, perpendicular width). Finally, the tumor burden was assessed by bioluminescence imaging with non-invasive IVIS Spectrum Imaging System (Perkin Elmer). Mice were killed then tumors were extracted and imaged.

### Ki67 immunostaining assay

Mice tumor sections were fixed in 4% paraformaldehyde. Paraffin embedded 5 μm sections were made for hematoxylin and eosin and IHC staining. We added citric acid buffer for antigen retrieval at 120 °C. Sections were blocked using PBS–H_2_O_2_ with 0.1% Tween-20. Ki67 antibody was added for incubating at 4 °C overnight and then secondary antibodies were added as well. DAB color was developed with diaminobenzene and hematoxylin. Stained slides were pictured with a microscopic.

### Statistical analyses

Each experiment was repeated three times and the data were shown as mean ± SD. Categorical variables were expressed as percentages. The significance between groups was determined using the two-tailed Student’s *t* test or one-way analysis of variance. Relationship between ZNF280A expression and tumor characteristics in lung cancer patients with was analyzed using Mann–Whitney *U* analysis and Spearman grade correlation analysis. Statistical significance was calculated by SPSS 22.0 (IBM) and *P* value < 0.05 was considered statistically significant. Graphs were made using GraphPad Prism 6.01 (Graphpad Software).

## Supplementary information

Supplementary figure legends

Figure S1

Figure S2

Figure S3

Figure S4

Figure S5

Figure S6

Table S1

Table S2

Table S3

Table S4
