# Supplementary material for: ZNF280A promotes lung adenocarcinoma development by regulating the expression of EIF3C
Source: Cell Death Dis. 2021 Jan 4;12(1):39. doi: 10.1038/s41419-020-03309-9 (PMC7791122; doi:10.1038/s41419-020-03309-9)
Supplement: Supplementary file 1 — Supplementary figure legends [file 41419_2020_3309_MOESM1_ESM.docx]

**Figure S1.** The transfection efficiencies of shZNF280A and shCtrl in A549 and NCI-H1299 cells were evaluated through observing the fluorescence of GFP on lentivirus vector.

**Figure S2.** Human Apoptosis Antibody Array was performed to detect and compare the expression of apoptosis-related proteins in NCI-H1299 cells with or without ZNF280A knockdown.

**Figure S3.** (A) A PrimeView Human Gene Expression Array was performed to identify the differentially expressed genes (DEGs) between shZNF280A and shCtrl groups of NCI-H1299 cells. (B) The volcano plot of gene expression profiling in NCI-H1299 cells with or without ZNF280A knockdown. Green dots represent the downregulated DEGs, red dots represent the upregulated DEGs. (C) The enrichment of the DEGs in canonical signaling pathways was analyzed by IPA. (D) The enrichment of the DEGs in IPA disease and function was analyzed by IPA.

**Figure S4.** (A) The knockdown efficiencies of 3 shRNAs prepared for silencing EIF3C were evaluated through qPCR. (B) The transfection efficiencies of shEIF3C and shCtrl in NCI-H1299 cells were evaluated through observing the fluorescence of GFP on lentivirus vector. ***P* < 0.01

**Figure S5.** (A) The transfection efficiencies of Control plasmid and ZNF280A overexpression plasmid were evaluated through observing the fluorescence of GFP on lentivirus vector. (B, C) The overexpression of ZNF280A in NCI-H1299 was confirmed by qPCR (B) and western blotting (C), respectively. ****P* < 0.001

**Figure S6.** (A) The transfection efficiencies of NC(OE+KD) and ZNF280A+shEIF3C in NCI-H1299 cells were evaluated through observing the fluorescence of GFP on lentivirus vector. (B) The mRNA and protein levels of ZNF280A and EIF3C in NCI-H1299 cells transfected with different plasmids were detected by qPCR and western blotting, respectively. Data was shown as mean ± SD. **P* < 0.05, ***P* < 0.01
