# Supplementary figures and images for: ZNF280A promotes lung adenocarcinoma development by regulating the expression of EIF3C
Source: Cell Death Dis. 2021 Jan 4;12(1):39. doi: 10.1038/s41419-020-03309-9 (PMC7791122; doi:10.1038/s41419-020-03309-9)

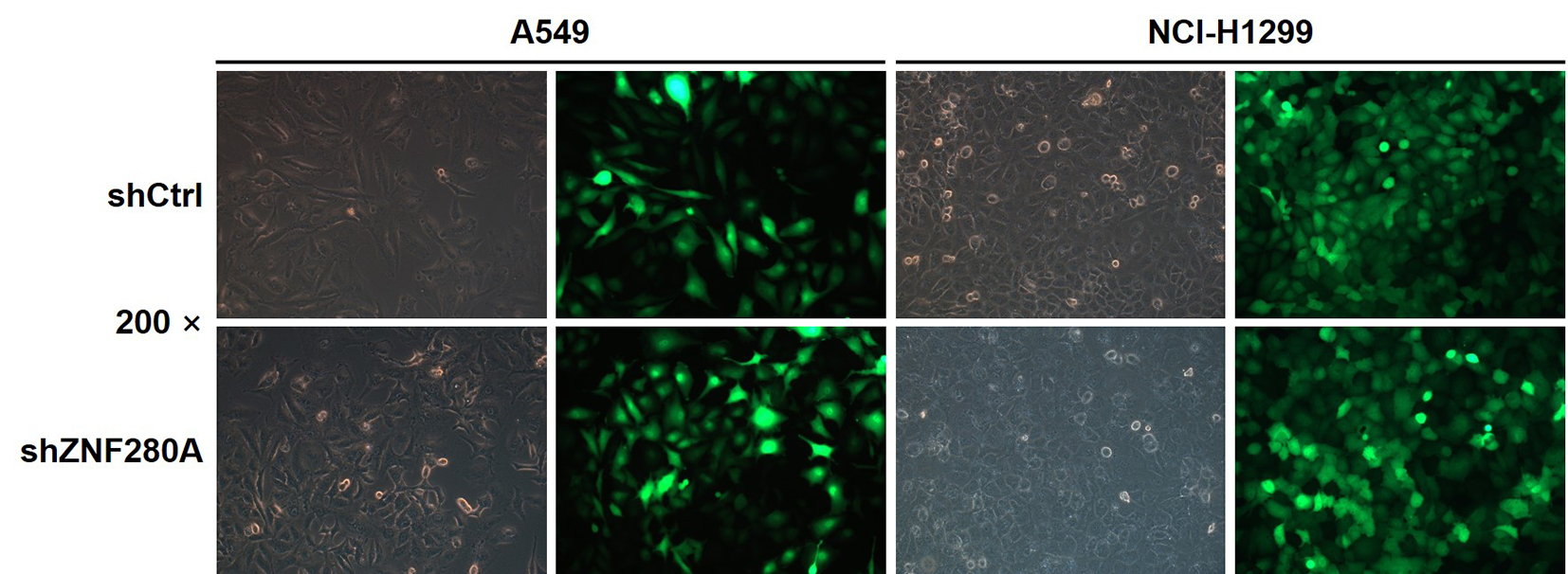

Supplement: Supplementary file 2 — Figure S1 [file 41419_2020_3309_MOESM2_ESM.tif]

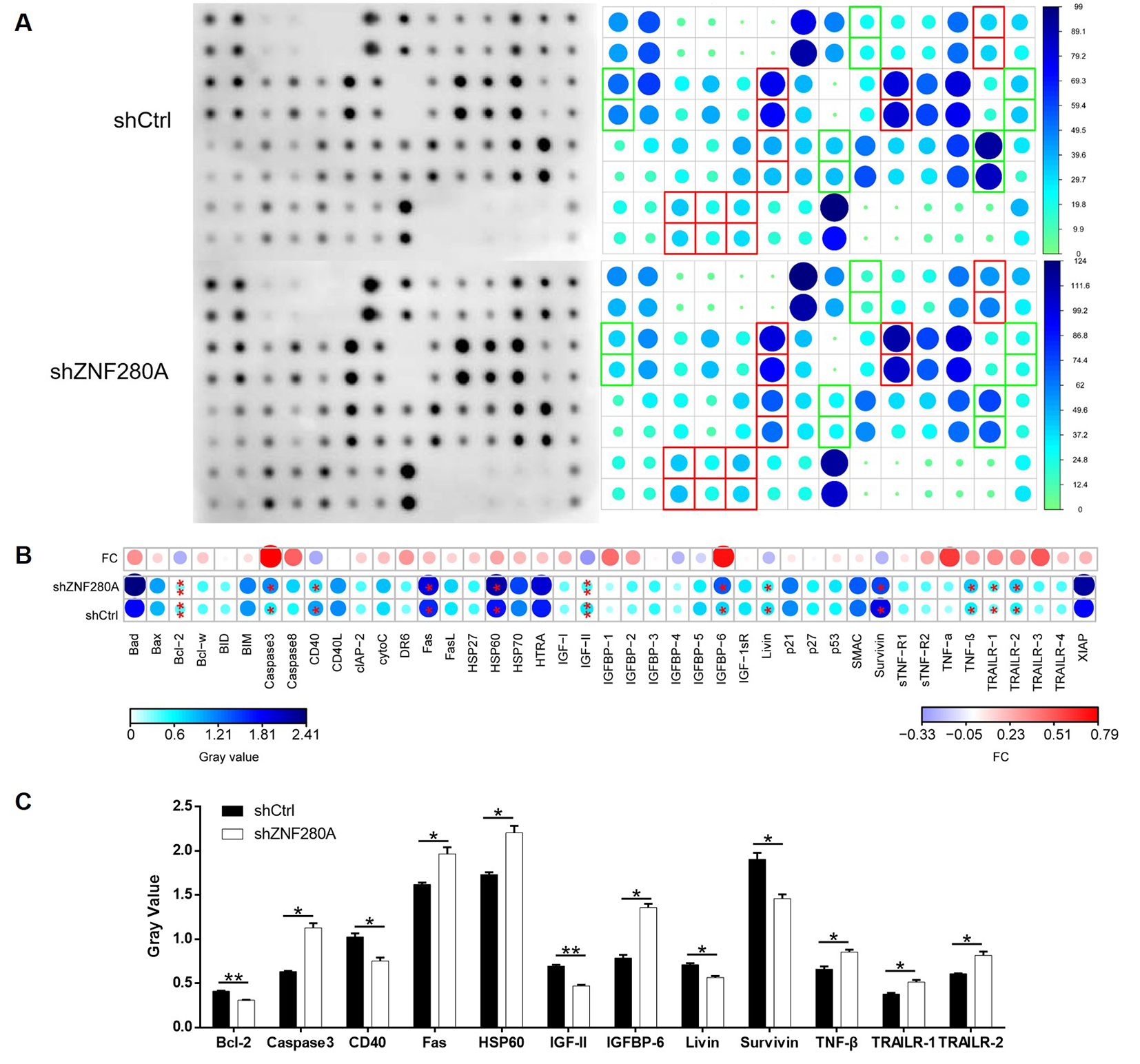

Supplement: Supplementary file 3 — Figure S2 [file 41419_2020_3309_MOESM3_ESM.tif]

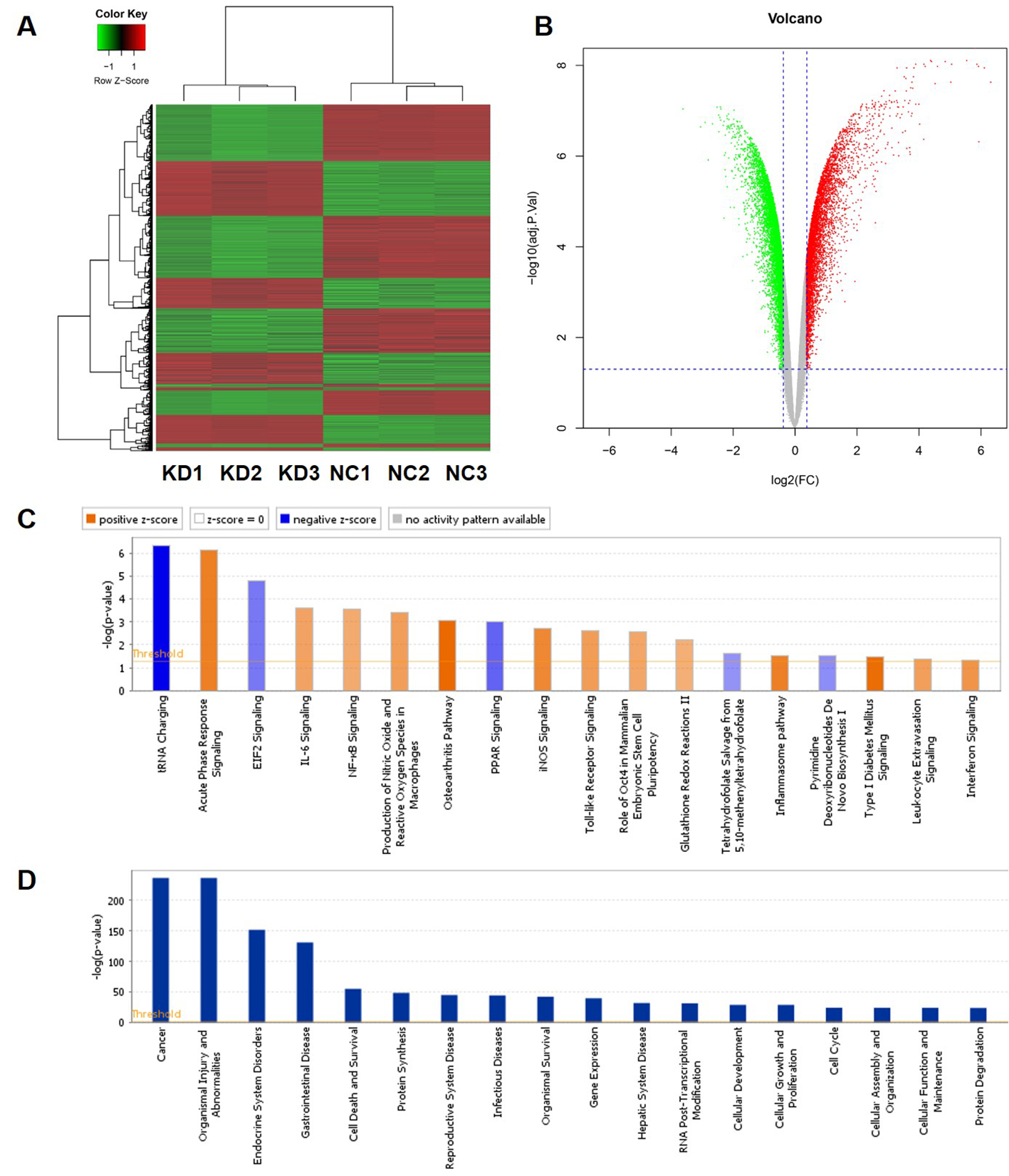

Supplement: Supplementary file 4 — Figure S3 [file 41419_2020_3309_MOESM4_ESM.tif]

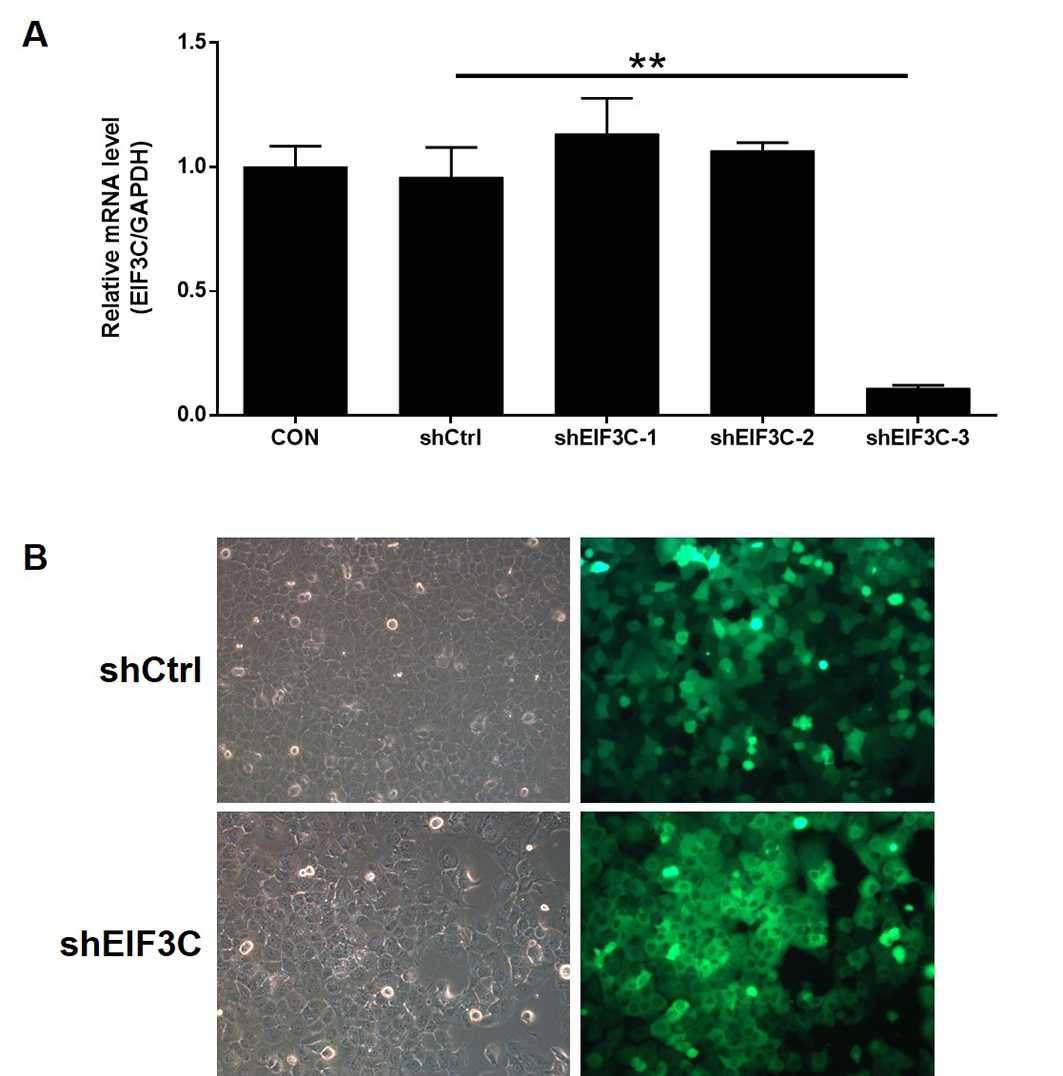

Supplement: Supplementary file 5 — Figure S4 [file 41419_2020_3309_MOESM5_ESM.tif]

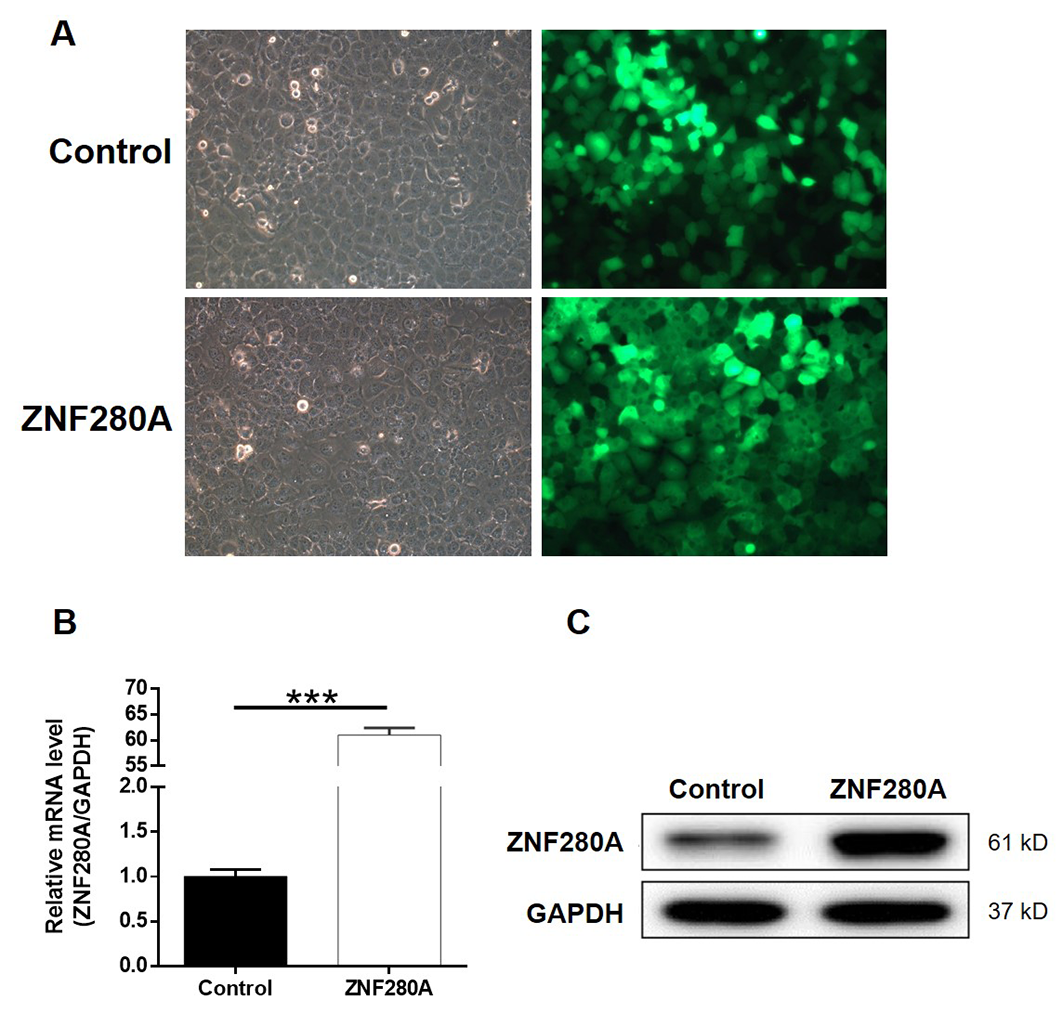

Supplement: Supplementary file 6 — Figure S5 [file 41419_2020_3309_MOESM6_ESM.tif]

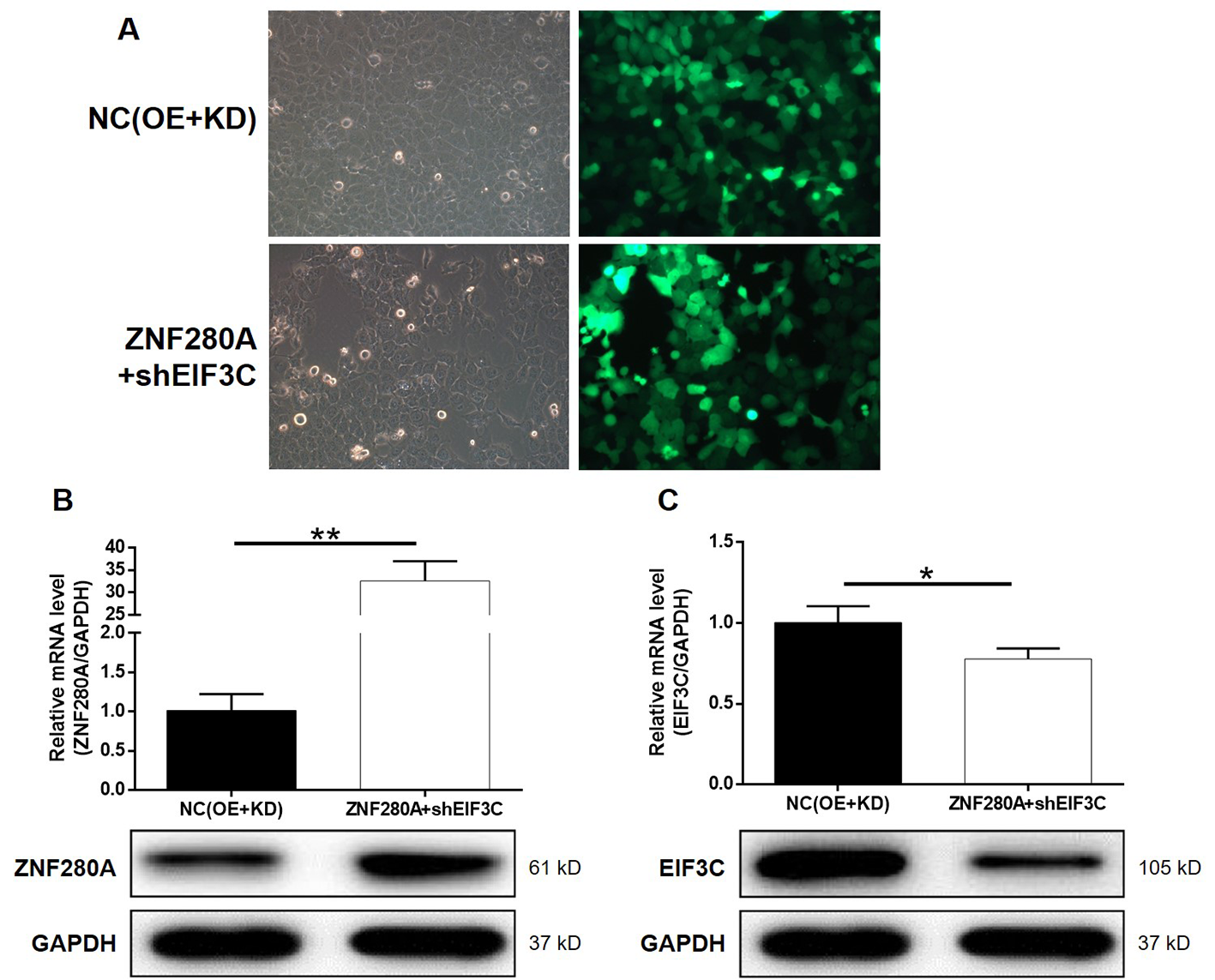

Supplement: Supplementary file 7 — Figure S6 [file 41419_2020_3309_MOESM7_ESM.tif]
