# Supplementary material for: ZNF280A promotes lung adenocarcinoma development by regulating the expression of EIF3C
Source: Cell Death Dis. 2021 Jan 4;12(1):39. doi: 10.1038/s41419-020-03309-9 (PMC7791122; doi:10.1038/s41419-020-03309-9)
Supplement: Supplementary file 8 — Table S1 [file 41419_2020_3309_MOESM8_ESM.docx]

Table S1 Antibodies used in western blotting and IHC

| Primary antibodies | Dilution in WB | Source species | Company | Catalog No. |
| --- | --- | --- | --- | --- |
| ZNF280A | 1:1000 | Mouse | abcam | ab169117 |
| GAPDH | 1:3000 | Rabbit | Bioworld | AP0063 |
| CDK1 | 1:2000 | Rabbit | Abcam | ab133327 |
| Cyclin D1 | 1:2000 | Rabbit | CST | 2978 |
| Cyclin E2 | 1:2000 | Rabbit | Abcam | ab40890 |
| EIF3C | 1:2000 | Rabbit | Abcam | ab237757 |
| RPL35A | 1:500 | Rabbit | biorbyt | orb513214 |
|  |  |  |  |  |
| Primary antibodies | Dilution in IHC | Source species | Company | Catalog No. |
| ZNF280A | 1:400 | Rabbit | Bioss | bs-12839R |
| Ki67 | 1:200 | Rabbit | abcam | ab6721 |
| EIF3C | 1:100 | Rabbit | abcam | ab19360 |
|  |  |  |  |  |
|  |  |  |  |  |
| Secondary antibody | Dilution |  | Company | Catalog No. |
| HRP Goat Anti-Rabbit IgG (WB) | 1:3000 |  | Beyotime | A0208 |
| HRP Goat Anti-Mouse IgG (WB) | 1:3000 |  | Beyotime | A0216 |
| HRP Goat Anti-Rabbit IgG (IHC) | 1:200 |  | Abcam | Ab111909 |
