# Supplementary material for: ZNF280A promotes lung adenocarcinoma development by regulating the expression of EIF3C
Source: Cell Death Dis. 2021 Jan 4;12(1):39. doi: 10.1038/s41419-020-03309-9 (PMC7791122; doi:10.1038/s41419-020-03309-9)
Supplement: Supplementary file 9 — Table S2 [file 41419_2020_3309_MOESM9_ESM.docx]

Table S2 The target sequences and shRNA sequences

| Gene | No. | Target sequence (5'-3') | shRNA sequences (5'-3') |
| --- | --- | --- | --- |
| ZNF280A | Pbr10330-A | CTGTCACTATGAAGTCTTCAT | ccggCTGTCACTATGAAGTCTTCATctcgagATGAAGACTTCATAGTGACAGtttttg |
| ZNF280A | Pbr103300-B | CTGTCACTATGAAGTCTTCAT | aattcaaaaaCTGTCACTATGAAGTCTTCATctcgagATGAAGACTTCATAGTGACAG |
| EIF3C | Pbr12670-A | AGAGTTTGAGCTCCTGGGAAA | CcggAGAGTTTGAGCTCCTGGGAAActcgagTTTCCCAGGAGCTCAAACTCTTTTTTg |
| EIF3C | Pbr12670-B | AGAGTTTGAGCTCCTGGGAAA | aattcaaaaaAGAGTTTGAGCTCCTGGGAAActcgagTTTCCCAGGAGCTCAAACTCT |
| EIF3C | Pbr12671-A | GAACGAATGGATGAAGAATTT | CcggGAACGAATGGATGAAGAATTTctcgagAAATTCTTCATCCATTCGTTCTTTTTg |
| EIF3C | Pbr12671-B | GAACGAATGGATGAAGAATTT | aattcaaaaaGAACGAATGGATGAAGAATTTctcgagAAATTCTTCATCCATTCGTTC |
| EIF3C | Pbr12672-A | CCTGGCTGACCTAGAGGACTA | CcggCCTGGCTGACCTAGAGGACTActcgagTAGTCCTCTAGGTCAGCCAGGTTTTTg |
| EIF3C | Pbr12672-B | CCTGGCTGACCTAGAGGACTA | aattcaaaaaCCTGGCTGACCTAGAGGACTActcgagTAGTCCTCTAGGTCAGCCAGG |
|  |  |  |  |
|  |  |  |  |
|  |  |  |  |
|  |  |  |  |
|  |  |  |  |
|  |  |  |  |
|  |  |  |  |
|  |  |  |  |
|  |  |  |  |
|  |  |  |  |
|  |  |  |  |
|  |  |  |  |
|  |  |  |  |
|  |  |  |  |
|  |  |  |  |
|  |  |  |  |
|  |  |  |  |
|  |  |  |  |
|  |  |  |  |
|  |  |  |  |
|  |  |  |  |
|  |  |  |  |
|  |  |  |  |
|  |  |  |  |
|  |  |  |  |
|  |  |  |  |
|  |  |  |  |
|  |  |  |  |
|  |  |  |  |
|  |  |  |  |
|  |  |  |  |
|  |  |  |  |
|  |  |  |  |
|  |  |  |  |
|  |  |  |  |
|  |  |  |  |
|  |  |  |  |
|  |  |  |  |
|  |  |  |  |
|  |  |  |  |
|  |  |  |  |
|  |  |  |  |
|  |  |  |  |
|  |  |  |  |
|  |  |  |  |
|  |  |  |  |
|  |  |  |  |
|  |  |  |  |
|  |  |  |  |
|  |  |  |  |
|  |  |  |  |
|  |  |  |  |
