# Supplementary material for: ZNF280A promotes lung adenocarcinoma development by regulating the expression of EIF3C
Source: Cell Death Dis. 2021 Jan 4;12(1):39. doi: 10.1038/s41419-020-03309-9 (PMC7791122; doi:10.1038/s41419-020-03309-9)
Supplement: Supplementary file 10 — Table S3 [file 41419_2020_3309_MOESM10_ESM.docx]

Table S3 Primers used in qPCR

| Gene | Forward primer sequence (5’-3’) | Reverse primer sequence (5’-3’) |
| --- | --- | --- |
| ZNF280A | GATCTGATCTATGTTGGGGTGGA | CGTGAGCAGGATATTGACGGA |
| VEGFA | CTTGCCTTGCTGCTCTACCT | TTCGTGATGATTCTGCCCTC |
| MYC | TGCTGCCAAGAGGGTCAAGT | GCTCCGTTTTAGCTCGTTCC |
| ATF3 | AGGATTTTGCTAACCTGACGC | ACCTCGGCTTTTGTGATGG |
| E2F6 | CTCCAGCAGAAACCAGATTGG | CCCGACACCTTCAGACCTTT |
| E2F4 | CTGGTGAACAAGGAGGCATG | GCAGAGGTGGAGGTGTAGAAAC |
| PPP1R15A | GCCCAGAAACCCCTACTCAT | GACAGCCAGGAAATGGACAG |
| CCNE2 | AGCTGGTCTGGCGAGGTTTT | GGCCTGGATTATCTGGGCTTC |
| DDIT3 | GAGCTGGAAGCCTGGTATGA | AGAAGCAGGGTCAAGAGTGGT |
| ZNF280B | CCTGGTACTAGCTGACTTCCTGT | GCTGTGGCTGAGAATATGGC |
| E2F7 | GCTCGCTATCCAAGTTATCCC | CATAGATGCGTCTCCTTTCCAC |
| PRPF3 | GACCAAACGAAACCTACACCA | TTCCCTCTTCACAGCACGAA |
| CDK4 | CTACCAGATGGCACTTACACCC | GCAAAGATACAGCCAACACTCC |
| MAGOH | TTCAGGCTCGGTTGTCTTTT | AAACTCAAACTCCAGGAACTCG |
| CDKN1B | AGGAATAAGGAAGCGACCTGC | TGGGGAACCGTCTGAAACAT |
| RBM42 | GGAACGCTTGAAGGAAATGG | TGCATCGCTTCTACTGTGGG |
| E2F3 | CCAAGGGCAAAGGAAGAGCT | AATACCCCATCGGGTGACTG |
| SNRNP200 | TTGTGCTCCAAGCTGACCGT | CGTTGAGCCTTGTCTCCCATA |
| CCND1 | AGCTGTGCATCTACACCGAC | GAAATCGTGCGGGGTCATTG |
| EIF3C | CAGAAAATGAGGGCGAGGAC | TAGATGTGGCAGAGGATGGC |
| RPL35A | GAAGGTGTTTACGCCCGAGAT | CGAGTTACTTTTCCCCAGATGAC |
|  |  |  |
