# Supplementary material for: ZNF280A promotes lung adenocarcinoma development by regulating the expression of EIF3C
Source: Cell Death Dis. 2021 Jan 4;12(1):39. doi: 10.1038/s41419-020-03309-9 (PMC7791122; doi:10.1038/s41419-020-03309-9)
Supplement: Supplementary file 11 — Table S4 [file 41419_2020_3309_MOESM11_ESM.docx]

Table S4 Relationship between ZNF280A expression and tumor characteristics in patients with lung cancer analyzed by Spearman rank correlation analysis

| Tumor characteristics | index |  |
| --- | --- | --- |
| Grade | Pearson correlation | 0.353 |
|  | Significance (two tailed) | 0.001 |
|  | n | 92 |
| Stage | Pearson correlation | 0.273 |
|  | Significance (two tailed) | 0.011 |
|  | n | 87 |
| T Infiltrate | Pearson correlation | 0.217 |
|  | Significance (two tailed) | 0.037 |
|  | n | 92 |
| Lymphatic  [metastasis](D:/360%E5%AE%89%E5%85%A8%E6%B5%8F%E8%A7%88%E5%99%A8%E4%B8%8B%E8%BD%BD/Dict/8.4.0.0/resultui/html/index.html#/javascript:;) (N) | Pearson correlation | 0.283 |
|  | Significance (two tailed) | 0.014 |
|  | n | 74 |
| Lymph node positive | Pearson correlation | 0.291 |
|  | Significance (two tailed) | 0.006 |
|  | n | 89 |
